# Simplified plasmid cloning with a universal MCS design and bacterial in vivo assembly

**DOI:** 10.1186/s12896-021-00679-6

**Published:** 2021-03-15

**Authors:** Fan Chen, Yi-ya Li, Yan-li Yu, Jie Dai, Jin-ling Huang, Jie Lin

**Affiliations:** School of Biological Science and Biotechnology, Minnan Normal University, Zhangzhou, 363000 P.R. China

**Keywords:** Plasmid cloning, Universal MCS, Bacterial in vivo assembly, DNA assembly, Homologous sequence

## Abstract

**Background:**

The ability to clone DNA sequences quickly and precisely into plasmids is essential for molecular biology studies. The recent development of seamless cloning technologies has made significant improvements in plasmid construction, but simple and reliable tools are always desirable for time- and labor-saving purposes.

**Results:**

We developed and standardized a plasmid cloning protocol based on a universal MCS (Multiple Cloning Site) design and bacterial in vivo assembly. With this method, the vector is linearized first by PCR (Polymerase Chain Reaction) or restriction digestion. Then a small amount (10 ~ 20 ng) of this linear vector can be mixed with a PCR-amplified insert (5× molar ratio against vector) and transformed directly into competent *E. coli* cells to obtain the desired clones through in vivo assembly. Since we used a 36-bp universal MCS as the homologous linker, any PCR-amplified insert with ~ 15 bp compatible termini can be cloned into the vector with high fidelity and efficiency. Thus, the need for redesigning insert-amplifying primers according to various vector sequences and the following PCR procedures was eliminated.

**Conclusions:**

Our protocol significantly reduced hands-on time for preparing transformation reactions, had excellent reliability, and was confirmed to be a rapid and versatile plasmid cloning technique. The protocol contains mostly mixing steps, making it an extremely automation-friendly and promising tool in modern biology studies.

**Supplementary Information:**

The online version contains supplementary material available at 10.1186/s12896-021-00679-6.

Plasmid cloning is one of the most commonly used techniques in molecular biology research. It plays a crucial role in studying the structure, function, and evolution of genes [1, 2] while serving as an essential tool in genetic, protein, and metabolic engineering [3, 4]. However, the traditional digestion-ligation method is often limited, as both vector and target fragments must have compatible cleavage sites. Moreover, the whole procedure can be time-consuming and labor-intensive [5].

To overcome the difficulties mentioned above, new alternatives have emerged in the past two decades. These include PCR-mediated IVA (In Vivo Assembly) cloning [6], fast cloning [7]; LIC (Ligation-Independent Cloning) such as ELIC (Exonuclease and Ligation-Independent Cloning) [8], SLIC (Sequence and Ligation-Independent Cloning) [9], HAC (Homologous Alignment Cloning) [10], One-step SLIC [11] and In-Fusion cloning [12]; recombination-based Gateway Cloning [13], SLiCE (Seamless Ligation Cloning Extract) [14, 15], and multienzyme-based Gibson Assembly [16], Golden Gate Assembly [17]. Each of these methods has advantages and limitations (more information is listed in Supplementary Table S1). For example, PCR-mediated and LIC methods remove a few enzymatic treatment steps. Nevertheless, multiple rounds of PCR amplification are required, which might result in random mutations in the final products. Recombination- and multienzyme-based methods are often used in commercial cloning kits. They are methods with excellent convenience, but the cost of applying these kits is also much higher. Furthermore, most cloning methods are not automation friendly because multiple pre-transformation steps or different primer designs are necessary for various vectors and inserts.

The *recA*-independent recombination pathway in *E. coli* (Fig. 1) has been reported for more than two decades [18, 19]. Although the mechanism of such recombination remains elusive, it was recently considered a useful tool for simplifying molecular cloning. When applying such methods, terminal sequences of linearized vectors were always chosen as the homologous linker required by in vivo assembly. Thus, as the vector sequence changes, the primers used to amplify the insert will have to be redesigned and synthesized, resulting in additional costs and labor.

**Fig. 1:**
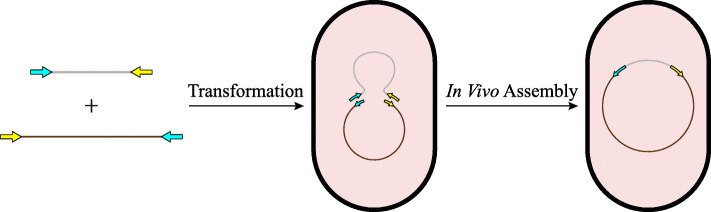
Schematic representation of bacterial in vivo assembly. Vector and insert molecules sharing the same homologous linker regions can be transformed into cells and then recombined to form a circular plasmid through in vivo assembly

In this study, inspired by previous reports [6, 8, 20, 21], we developed a novel cloning strategy based on a UMCS (universal MCS) design and bacterial in vivo assembly. The method uses 10 ~ 20 ng of linearized vector and a 5× molar ratio of insert fragment for successful subcloning with high efficiency and fidelity. The whole transformation process can be performed within 1.5 h when carried out using classic chemically component cell strains. The main concept of our strategy is to design and synthesize a universal MCS that can be used to replace the existing MCS of each vector. When the UMCS serves as the homologous linker, the primers to amplify the vector are determined. Only the template binding section of primers for insert amplification needs to be changed. Due to the consistency of homologous linker sequences, the same amplified DNA fragments can be cloned into any modified vector with the UMCS without extra PCR steps. This makes the method simple, robust, highly versatile, and further meets the requirements of a high-throughput automated operation [22].

## Results

### Universal MCS and its associated primer design

**Fig. 2:**
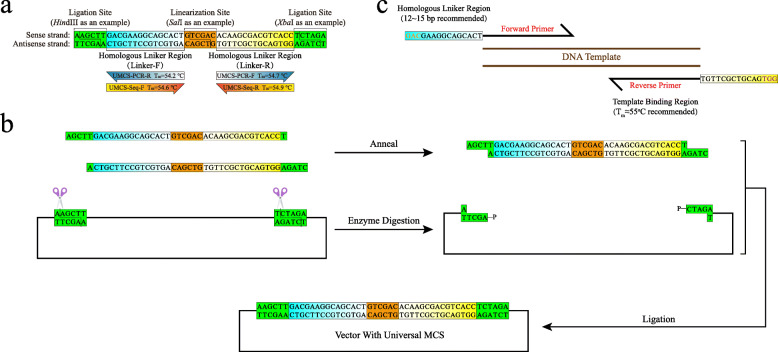
Principle of the UMCS and corresponding primer design. **a** The UMCS sequence used in this study contains two homologous linker regions that are connected by the *Sal*I recognition sequence. The flanking *Hind*III and *Xba*I recognition sequences were designed for replacing the original MCS. All three recognition sites can be freely substituted for other sequences. **b** The UMCS was first synthesized as two ssDNAs (each with a length of 42 bp). After annealing and ligating, the UMCS was incorporated into the vector, thus replacing the original MCS. **c** The recommended primer design to maximize assembly efficiency. The homologous linker region (12 ~ 15 bp) is included in the 5′-terminus of each primer, and the T_m_ of the template binding region was designed to be approximately 55 °C

The design of the UMCS for universal subcloning is shown in Fig. 2a. The main features of this novel MCS design are as follows: (1) The length of UMCS is only 48 bp, which is shorter than many of the existing MCSs. The actual sequence to be synthesized is 42 bp for both the forward and reverse directions (Fig. 2b). By annealing and ligating to a predigested vector molecule, the plasmid with the UMCS was reconstituted. (2) The restriction enzyme recognition sites at both termini are needed only for UMCS integration. They can be replaced freely according to the original MCS compatibility of the vector. (3) The restriction enzyme recognition site in the middle of the UMCS is used only for vector linearization, which can be substituted with any other sequences for digestion if they are compatible with the vector. (4) The two homologous linker regions were carefully optimized to have close T_m_ and the same GC content, which are supposed to facilitate PCR. Hairpin and dimerization of these regions were also minimized through optimization studies. (5) Primers used for the amplification of the insert can be easily designed by introducing a homologous linker at their 5′-terminus, while the T_m_ of the template binding region is calculated to be approximately 55 °C (Fig. 2c). (6) Theoretically, because of MCS consistency, any vector with a UMCS sequence can be amplified by the “UMCS-PCR-F + UMCS-PCR-R” primer pair, and any insert cloned into the UMCS can be amplified by the “UMCS-Seq-F + UMCS-Seq-R” primer pair. (7) Both linker regions do not contain rare codons [23, 24]. If the linker sequence is to be expressed, the codons contained in the UMCS are unlikely to affect the target protein expression. This is crucial for vectors that express N-terminal tags.

### Construction of pUC19-UMCS-EGFP

**Fig. 3:**
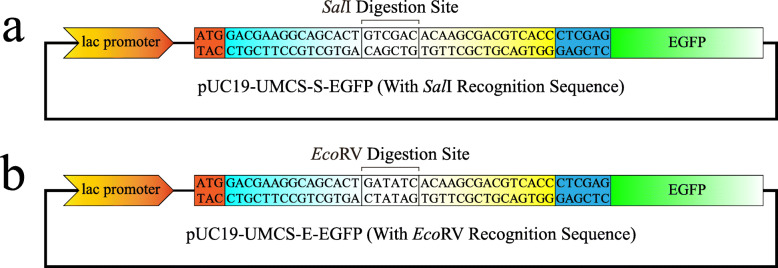
Schematic depicting of pUC19-based plasmid models. Two different designs of the pUC19-UMCS-EGFP plasmid with *Sal*I or *Eco*RV recognition sequence were used for sticky- or blunt-end digestion

To demonstrate proof of principle, we first constructed two types of pUC19-UMCS-EGFP vectors (Fig. 3) as tools for studying the transformation efficiency. These vectors are modified versions of the pUC19 because the *lac*Z sequence was first replaced by UMCS-*Xho*I sequences with different linearization sites (*Sal*I or *Eco*RV, as shown in Fig. 3a and 3b), followed by the insertion of EGFP (Enhanced Green Fluorescent Protein) through *Xho*I-based digestion and ligation. Among them, *Sal*I represented the linearized product with sticky ends, while *Eco*RV represented blunt ends. Together with vectors linearized by PCR, the effect of different linearization methods on transformation can be clarified. During in vivo assembly, if the mCherry CDS is successfully cloned into the UMCS, the colony will appear orange. Otherwise, it will be green or colorless (through nonspecific assembly). By calculating orange colonies against the total number of colonies, the positive ratio of transformation can be determined.

### Effects of the linearization method and homologous linker length

**Fig. 4:**
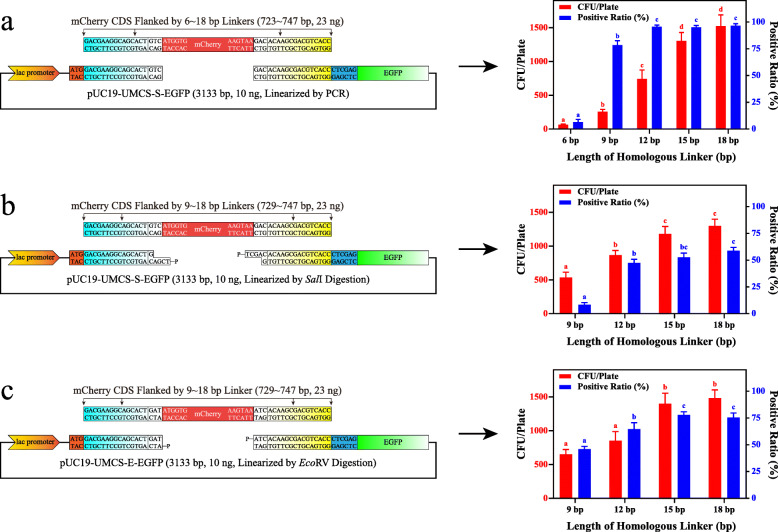
Effects of the linearization method and homologous length on assembly efficiency. **a** Transformation efficiency (shown as CFU/plate and positive ratio of colonies) of PCR-linearized pUC19-UMCS-S-EGFP with the mCherry sequence flanked by 6-, 9-, 12-, 15-, and 18-bp homologous linkers (*n* = 4). **b and c** Transformation efficiency of *Sal*I- and *Eco*RV-digested pUC19-UMCS-S-EGFP and pUC19-UMCS-E-EGFP with the mCherry sequence flanked by 9-, 12-, 15-, and 18-bp homologous linkers (*n* = 4)

Next, we evaluated the effects of different linearization methods and the length of the homologous linker in terms of the number of transformants and the corresponding positive ratio. As shown in Fig. 4a, when PCR was used as a linearization method, the transformation efficiency showed a positive correlation with the length of the linker sequence. When a 6-bp linker was used, less than 100 CFU/plate and a positive ratio below 10% were confirmed. In contrast, the efficiency of transformation increased to 259 ± 34, 742 ± 132, 1306 ± 123, and 1525 ± 165 CFU/plate, and the positive ratio increased dramatically to 78 ± 4.2%, 96 ± 1.5%, 95 ± 1.7%, and 97 ± 1.9% when 9-, 12-, 15-, and 18-bp linkers were tested, respectively. It is evident that a 6-bp linker is not enough for bacterial in vivo assembly, and a length longer than 9 bp is necessary when performing such experiments. Moreover, there were no significant differences between the 15- and 18-bp linker. Considering both the colony number and positive ratio, we concluded that the length of the homologous linker for the PCR method was 12 ~ 15 bp. On the other hand, when linearization was carried out by restriction digestion, it was shown (Fig. 4b and 4c) that the digested vectors with blunt ends (*Eco*RV) yielded much more positive clones than sticky ends at any of the linker lengths we tested. According to the data collected, when a restriction enzyme was used for linearization, a 12 ~ 15-bp linker also ensured enough transformants and positive clones to screen. In these cases, further increase the linker length is not necessary.

### Effects of insert/vector ratio on transformation

**Fig. 5:**
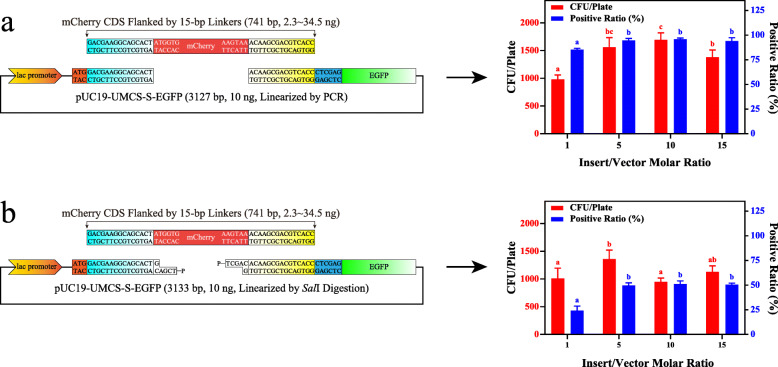
Effects of insert/vector ratio on assembly efficiency. Effects of insert/vector molar ratio on the recombination of pUC19-UMCS-S-EGFP linearized by PCR (**a**) or *Sal*I digestion (**b**) with the mCherry sequence flanked by 15-bp homologous linkers (*n* = 4)

The molar ratio of insert/vector is considered a critical factor for high-efficiency transformation [25]. Thus, we further investigated the influence of PCR, *Sal*I and *Eco*RV digestion on transformation efficiency at multiple insert/vector levels. The yields of transformants and the positive ratio at the linker length of 15 bp were determined (Fig. 5 and Supplementary Figure S1) with molar ratios of 1, 5, 10, and 15. According to the data shown, regardless of the linearization method used, the optimal molar ratio remained at 5. A further increase in insert usage will not improve the results, while a decreased insert quantity shows a negative effect on transformation efficiency. Therefore, we used a molar ratio of 5 in all subsequent studies, which is consistent with the finding of Kostylev et al. [26]

### Effects of the digestive linearization method on the fidelity of assembly

**Fig. 6:**
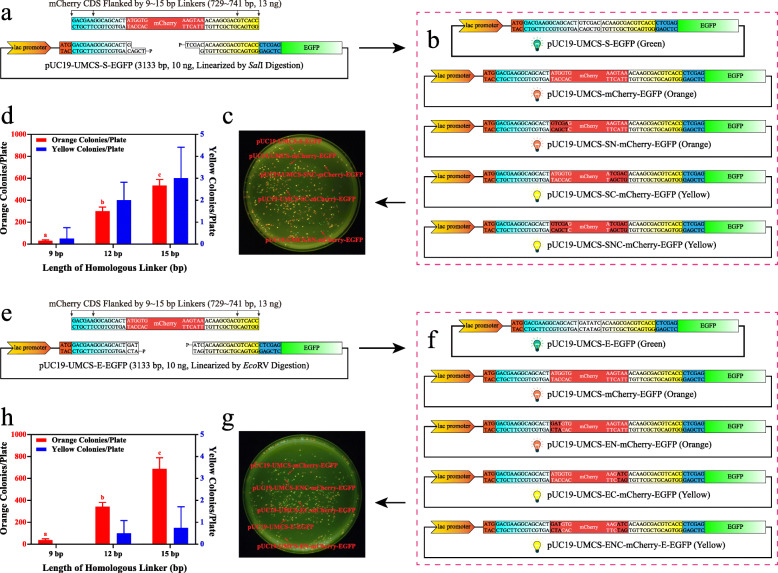
Assembly fidelity evaluation of vectors linearized by restriction digestion. **a** Schematic assembly of *Sal*I-digested pUC19-UMCS-S-EGFP and mCherry CDS with 9-, 12-, and 15-bp homologous linkers. **b** The five possible plasmid products that can be observed after the transformation of vectors and inserts mentioned in (**a**). Among them, if the 3′-terminus of mCherry CDS was replaced by part of the *Sal*I recognition sequence (“TCGAC”), the stop codon of the mCherry protein would then be destroyed, resulting in the expression of the mCherry-EGFP fusion protein with a characteristic yellow color that can be counted accurately. **c** Colonies with green, orange, and yellow colors can be observed when cells express different fluorescent proteins. Note that cells transformed with the pUC19 plasmid (colorless) were also spread onto the plate as a reference. **d** The orange and yellow colonies counted after the transformation of vectors and inserts mentioned in (**a**). Method fidelity can be calculated by one minus the number of yellow colonies divided by the number of orange colonies (*n* = 4). **e-h** The other experiments and results following the same procedure described in (**a-d**), except the vector was linearized by *Eco*RV digestion

We were interested in the assembly fidelity of the digestive linearization method mainly based on three reasons: (1) Although high fidelity enzymes such as Q5, Phusion, or KOD have extremely low mismatch rates [27, 28] during the reaction, PCR products could still suffer from random errors introduced by DNA polymerase. Moreover, the possibility of introducing random mutations during the PCR process increases with PCR cycles and the length of vectors, and it is laborious to verify by sequencing. (2) Some vectors are difficult to amplify by PCR. For example, the GC content of the vector is too high or too low, or the vector contains too many repetitive sequences, which will affect PCR and even render the reaction impossible to continue [29–31]. (3) As previously mentioned, if vectors cannot be linearized by PCR or fidelity is vital for the experiment, the methods shown in Fig. 6 must be applied. Under such circumstances, only the sequences flanking the linearization site are used as homologous linkers to ensure the versatility of UMCS. However, when the flanking sequences (15 bp) were used as homologous linkers, the post-digestion residual bases then served as nonhomologous sequences. This means the residual bases might displace part of the insert and cause mutations at its 5′-terminus, 3′-terminus (confirmed during our pilot study), or both. The frequency of such events is a major concern for UMCS applications, since the UMCS is only universal when this frequency is low enough.

Therefore, *Sal*I (Fig. 6a-d) and *Eco*RV (Fig. 6e-h) digestion were taken as sticky- and blunt-end examples to evaluate sequence replacement between insert and residual bases after assembly. As shown in Fig. 6, when the mCherry CDS was used as an insert, five possible products could be expected: (1) if the assembly fails, the colony will appear green (only EGFP expression); (2) if the fragments assembled successfully and the mCherry CDS remains intact, the colony will appear orange (only mCherry expression); (3) if assembled successfully, but the 5′-terminus of the mCherry CDS is partially replaced, the colony will appear orange (only mCherry expression); (4) if assembled successfully, but the 3′-terminus of the mCherry CDS is partially replaced, the colony appears yellow for the loss of the mCherry stop codon (mCherry-EGFP fusion protein is expressed); and (5) if assembled successfully, while both the 5′- and 3′-terminus of the mCherry CDS are partially replaced, the colony also appears yellow (mCherry-EGFP fusion protein is expressed). For subcloning, we assume that the 5′- and 3′-terminus of the insert will be replaced at the same frequency, then the frequency can be derived by calculating the number of yellow colonies against the orange ones. Thus, Fig. 6d strongly suggests that the frequency of mutation at the 3′-terminus of the insert is low when *Sal*I is used for vector linearization. In fact, less than 0.75% of positive clones contained sequence replacement at the 3′-terminus or at both the 5′- and 3′-terminus. This indicates that the possibility of having at least one mutated terminus is less than 1.5% when the length of the linker is 12 or 15 bp. Therefore, it will be very unlikely for anyone to pick up a mutated product by chance. Moreover, when *Eco*RV was used for linearization, the frequency of replacement events seemed less than that of *Sal*I by having a statistical value of less than 0.3% on both 12- and 15-bp linkers. In conclusion, after digestion, the residual bases slightly affected the fidelity of assembly (an assumed mechanism of bacterial in vivo assembly with nonhomologous regions is given in Supplementary Fig. S2), but most of the positive colonies were still expected to contain the correct plasmid.

### Construction of larger plasmid based on UMCS

**Fig. 7:**
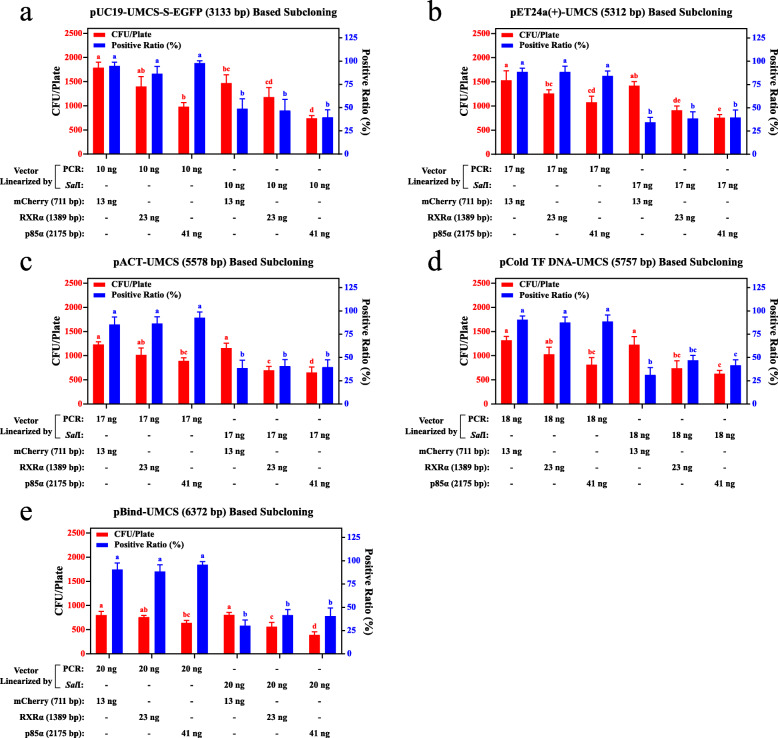
Additional versatility test for UMCS. a-e The efficiency of in vivo assembly according to 5 different UMCS-based vectors and 3 inserts was evaluated. All vector/insert combinations were repeated four times. For each transformation, 24 colonies were randomly picked for colony PCR and then subjected to agarose gel electrophoresis analysis. PCR products showing the same band as that of the insert were considered positive

To further demonstrate the versatility of the UMCS, we constructed an additional four vectors containing UMCS: (1) pET24a(+)-UMCS, the UMCS was inserted between the original *Bam*HI and *Xho*I restriction sites, resulting in a plasmid size of 5312 bp; (2) pACT-UMCS and pBind-UMCS, the UMCS was inserted between the original *Bam*HI and *Xba*I restriction sites, the last “C” of the *Bam*HI recognition sequence was intentionally removed to ensure the correct expression of the insert, resulting in the plasmid size of 5578 and 6372 bp; (3) pCold TF DNA-UMCS, the UMCS was inserted between the original *Nde*I and *Xba*I restriction sites, resulting in a plasmid size of 5757 bp. Together with the pUC19-UMCS-S-EGFP that was constructed previously, we examined the transformation efficiency of cloning the mCherry (711 bp), RXRα (Retinoid X Receptor Alpha, 1389 bp), and p85α (Phosphoinositide 3-kinase p85α, 2175 bp) CDS into these vectors. During these experiments, each CDS plus a 15-bp homologous linker region (as shown in Fig. 3a) at both termini was amplified as insert, each transformation was repeated four times, and colony PCR was performed on a panel of 24 colonies randomly selected from each transformation, followed by agarose gel electrophoresis analysis to identify the positive colonies and the positive ratio accordingly. The corresponding results are shown in Fig. 7. In summary, (1) regardless of the linearization method we tried, the yield of transformants decreased as the size of the final plasmid increased; (2) if PCR was used for vector linearization, no apparent correlations between the positive ratio and the size of the plasmid were observed, and the positive ratios were maintained at a high level (> 85%) in all experiments; and (3) when *Sal*I digestion was used, the positive ratio decreased with the increasing size of the final product. However, in our study, this ratio was always maintained at more than 1/3, which is enough for screening positive clones by PCR.

### Multi-fragment assembly

**Fig. 8:**
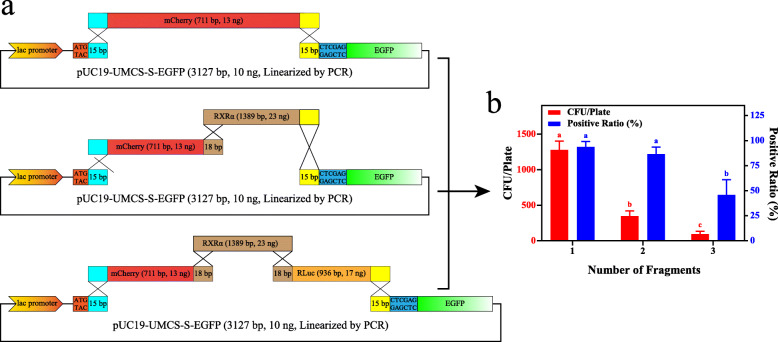
Multi-fragment assembly. **a** Schematic representation of multi-fragment assembly assessment. We used up to 3 different insert fragments for multi-fragment assembly experiments. Each insert has a 5× molar concentration of the PCR linearized vector. **b** Effects of the fragment count on transformation. The numbers of transformants and positive clones decreases with an increasing number of fragments. For each transformation, 24 colonies were randomly picked for colony PCR and then subjected to agarose gel electrophoresis analysis. PCR products showing the same band as that of the assembled insert(s) were considered positive

Similar to the single-fragment experiments, we used mCherry, RXRα, and RLuc (Renilla Luciferase) for multi-fragment assembly test. As presented in Fig. 8a, the 5′-terminus of the first fragment and the 3′-terminus of the last fragment must have homologous sequences corresponding to the UMCS. The linkers of other fragments needing to be assembled can be designed according to previous studies [6]. Since multi-fragment assembly significantly reduces the yield of transformants and positive clones (Fig. 8b), PCR as a linearization method or dephosphorylation after enzyme digestion is recommended. Meanwhile, further increasing the amount of DNA and the number of component cells might be necessary for the assembly of more than 3 insert fragments.

## Discussion

In most cases, inserts are required to be placed somewhere within a MCS to function properly. The UMCS-based cloning, like the MCS-based cloning, is not a seamless technique because it relies on synthetic homologous regions to work. However, it provides key advantages over current plasmid cloning methods:

First, we successfully constructed tens of plasmids with single or multiple insertions in a very efficient way by using this method. Although we used DH5α cells throughout this study, other strains such as Top10, BL21(DE3), XL-10 Gold and JM109(DE3) were also tested (as shown in Supplementary Figure S3 and Supplementary Table S2) and had similar success, highlighting the versatility of this protocol.

Second, the *Dpn*I enzyme, which is widely used in other reports [6, 7, 20, 26, 32] for the digestion of template DNA, is not necessary when using our protocol because only a few nanograms of linearized vector DNA and a 5× molar concentration of the insert fragment are needed. Thus, a single tube of carefully stored linearized vector or insert can be used several times without losing efficiency. Therefore, the cost of applying this protocol is well controlled.

Third, the UMCS protocol contains mostly mixing steps when both the vector and insert are ready, making it extremely automation friendly. Instruments for high-throughput transformations are much easier to design and build according to this approach, making this protocol promising in modern molecular biology studies.

Finally, and especially, because of the consistency of homologous linkers and the associated primers, any linearized UMCS-based vector and insert can be mixed directly and is ready for transformation. This is important for those who need to optimize protein expression or switch epitope tags by cloning the same insert into different vectors. In addition, the protocol uses only purified DNA, which means the high-fidelity DNA polymerases used to amplify vectors or inserts are expected to have little or no impact on the transformation. This further guarantees the versatility of UMCS because dedicated vectors or inserts are easier to share between researchers from laboratory to laboratory, regardless of their amplification conditions and without additional PCR steps.

In this study, by comparing different linearization methods, we found that even though the number of transformants was occasionally higher when using the digestion method, the yield of positive clones was more than 20% lower than that of PCR (Fig. 4). Interestingly, the DNA ligase activity inside *E. coli* has shown a great impact on the positive ratio. When comparing Fig. 4a and 4c, we found that when an 18-bp linker was used, the colonies formed after transformation were 1525 ± 165 and 1483 ± 121 without a significant difference, while the positive ratio was reduced significantly from 96.5 ± 1.9% to 75.4 ± 4.2%, implying the linearized vector with the phosphorylated 5′-terminus is more easily recircularized than the vector without phosphorylation. Additionally, recircularization by DNA ligase is more effective for vectors with sticky ends, which explains why the blunt-ended vector yields a higher positive ratio than the vector with sticky ends, and the PCR-amplified vectors work the best for in vivo assembly. Thus, if the vector itself is small enough or the fidelity of PCR is not a vital concern, PCR is a better choice for linearization, whereas if the vector is large enough that the random mutation introduced by PCR is problematic or the vector is simply resistant to PCR amplification, linearization with the blunt-end enzyme is also an effective strategy. Finally, if no suitable blunt-end enzymes’ recognition sequence could be found, digestion by sticky-end enzymes is still a feasible alternative. Furthermore, we believe that if restriction digestion was used for linearization, dephosphorylation might be helpful to further enhance transformation efficiency, especially for multi-fragment assembly [33].

**Fig. 9:**
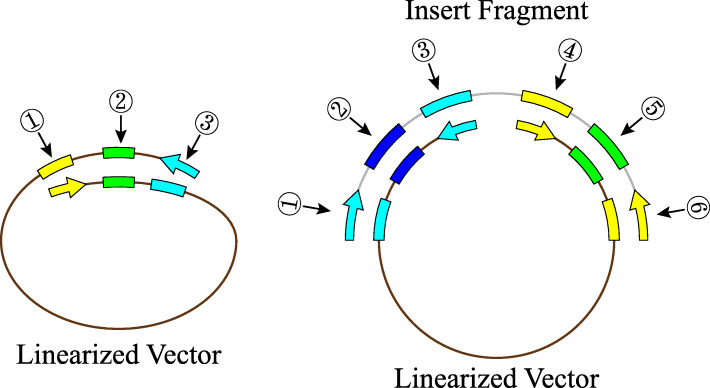
Proposed models for nonspecific assembly. Nonspecific assembly occurs within the vector molecule or between the vector and insert. Any of the marked assemblies (1 ~ 3 within vectors and 1 ~ 6 between vectors and inserts) occurred, a nonspecific assembly was confirmed

During our research, nonspecific assembly within vectors was observed occasionally. In rare cases, nonspecific assembly between the vector and insert could also be identified. As shown in Fig. 9, we have suggested three nonspecific assembly models within the vector molecule and six models between the vector and insert. When linearized pUC19-UMCS-S-EGFP or pUC19-UMCS-E-EGFP was involved, the results of nonspecific assembly usually appeared as nonfluorescent (the fluorescent protein or promoter sequence was lost or mutated) colonies. Such cases usually made up less than 5% of the total colonies, regardless of the linearization method. It was also reported previously [34] that the PCR products can be transformed directly and circularized within the cell. Therefore, nonspecific assembly and direct transformation of PCR products might be the two reasons for positive ratios seldom exceeding 97%, even when PCR products purified by agarose gel electrophoresis were used.

Conley et al. [35] proposed a mechanism for bacterial in vivo assembly: double-stranded linear DNA molecules are the substrate for 3′-5′-exonuclease action by ExoIII, which yields single-stranded homologous DNA sequences. After annealing at homologous sites, the vector and insert then circularize to form a plasmid molecule with gaps. The gaps are subsequently filled by DNA polymerase I and ligases as a DNA repair mechanism, thus completing plasmid formation. In this model, nonhomologous parts of the ssDNA termini will be displaced by newly-repaired duplex after recombination. However, it is hard to explain solely by this assumption why the intervening nonhomologous sequence (such as post-digestion residual bases) between homologous regions and linear DNA termini still exists as part of the final product in some cases. According to our experiments, such events happened with a probability of around 1.5 and 0.3% when using *Sal*I or *Eco*RV digestion, respectively. Although this phenomenon still favors us, how cells decide which sequence to keep within the final plasmid remains unclear. From this perspective, despite the significant improvements we have made upon understanding the recombination mechanism [36, 37], the picture is still far from complete.

**Fig. 10:**
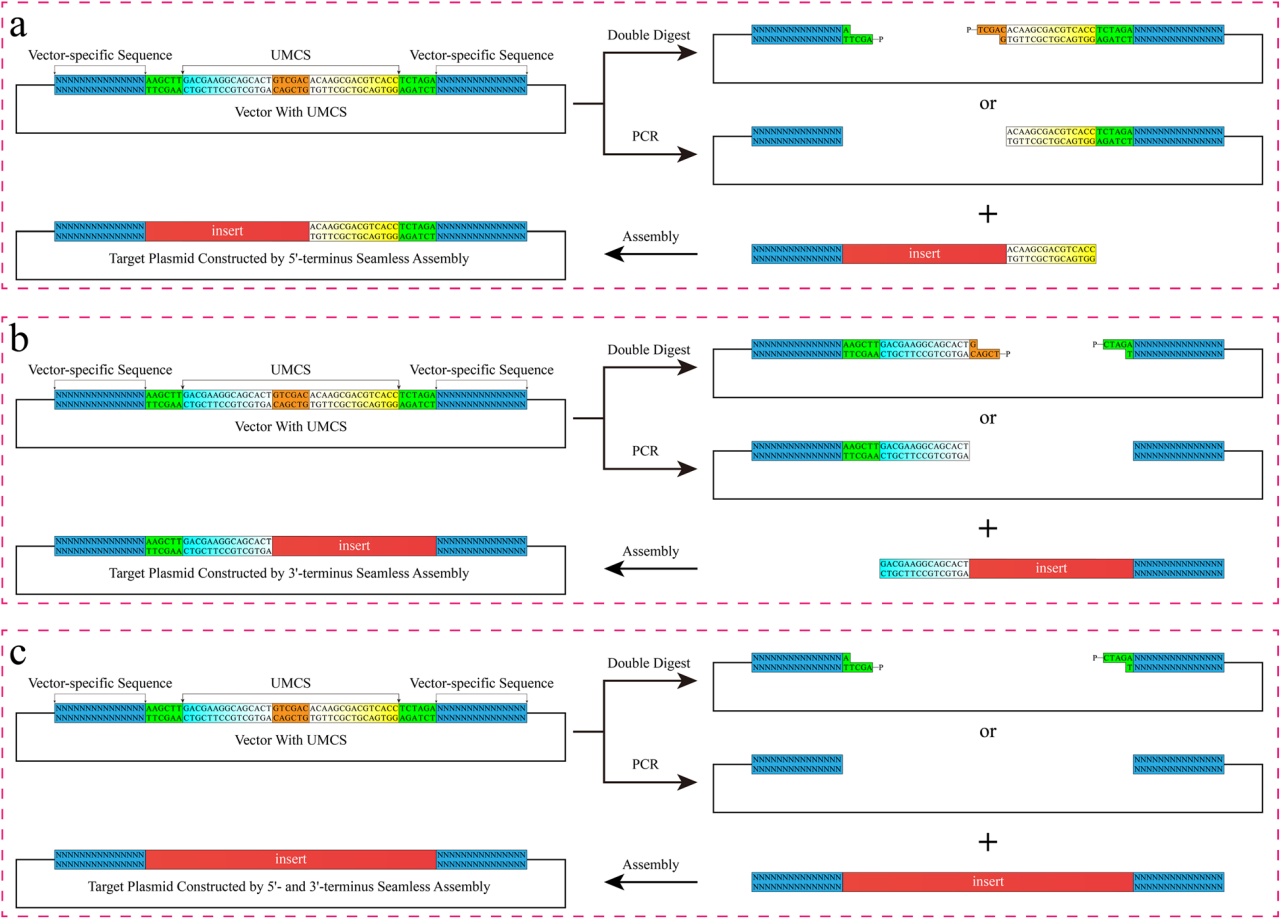
Seamless solutions for vectors with UMCS. **a-b** Alternative “half-seamless” flow chart of plasmid assembly using UMCS based vectors. **c** Schematic details of “full-seamless” assembly when UMCS must be ruled out of the final product

In some cases, the UMCS might affect the function or expression of the insert. For example, if the vector has a start codon located upstream of the UMCS (as shown in Fig. 3 and also typical in pET system for the expression of fusion proteins with N-terminal tags), the sequence of Linker-F (see Fig. 2 for detailed information) will be translated as a short peptide of “DEGST”. If such peptide impairs the insert’s function or expression, it must be removed when constructing the plasmid. The same strategy also applies when the expression of fusion proteins with C-terminal tags (stop codon located downstream of the UMCS) was affected by the sequence of Linker-R (translated as “TSDVT”). Consequently, alternative “half-seamless” or “full-seamless” strategies presented in Fig. 10 can be established. Among them, the “half-seamless” strategies (Fig. 10a and b) for 5′- or 3′-end seamless cloning still simplify the primer design, which will save time and cost of the experiments and reduce the system complexity.

## Conclusions

In this study, we developed and standardized a digestion-ligation-free approach for rapid and efficient plasmid cloning. The method utilizes a unified MCS design scheme, which simplifies primer design and the following PCR steps. Most importantly, it exploits the in vivo assembly of *E. coli* and significantly reduces hands-on time in the preparation of transformation to just seconds while maintaining excellent reliability.

## Materials and methods

### Bacterial strains and reagents

Chemically competent cell strains of *E. coli* DH5α, Top10, BL21(DE3), XL10-Gold, and JM109(DE3) were obtained from Weidi Biotechnology (Shanghai, China) with transformation efficiency of ~ 5 × 10^8^, ~ 5 × 10^8^, ~ 10^7^, ~ 2 × 10^9^, and ~ 10^8^ CFU/μg pUC19, respectively. Bacteria containing plasmids were cultured in LB (Luria-Bertani) medium with appropriate antibiotics (ampicillin or kanamycin at 100 or 50 μg/mL, respectively), and IPTG (Isopropyl β-D-1-thiogalactopyranoside, 100 μM) was added when necessary. The SanPrep Column Plasmid Mini-Preps Kit (Sangon Biotech, Shanghai, China) was used for plasmid extraction from bacteria, the SanPrep Column PCR Product Purification Kit (Sangon Biotech) was used for PCR product purification, and the SanPrep Column DNA Gel Extraction Kit (Sangon Biotech) was used for PCR product or enzyme-digested DNA purification after agarose gel electrophoresis, all following the manufacturer’s instructions. Both the 4S Red Plus (Sangon Biotech) stained 1% agarose gel and DNA Marker (250 ~ 10,000 bp, Sangon Biotech) were used for all gel electrophoresis experiments. FastDigest *Bam*HI, *Eco*RV, *Nde*I, *Sal*I, *Xba*I, *Xho*I, and T4 DNA ligase from Thermo Fisher Scientific (Shanghai, China) were used for vector construction or linearization. Q5® High-Fidelity DNA Polymerase from NEB (Ipswich, MA, USA) was used to amplify the vector and insert, while Taq PCR Master Mix (Sangon Biotech) was used for colony PCR.

### Primers and plasmids

Primers were designed using Oligo 7, the T_m_ of each primer was calculated according to the “Nearest Neighbor” method. All primers used in this study were designed then synthesized (as shown in Supplementary Table S3) and obtained from Sangon Biotech. The pUC19 plasmid was purchased from Weidi Biotechnology. The pET24a(+) vector was purchased from Invitrogen (Carlsbad, CA, USA). pCold TF DNA was purchased from Takara (Beijing, China). pACT and pBind were purchased from Promega (Madison, WI, USA). pCDNA3.1(+)-EGFP, pCDNA3.1(+)-mCherry, pCMV-RXRα and pCMV-p85α were all gifts from Xiaokun Zhang.

### PCR conditions and product analysis

Unless otherwise stated, 50 μL PCR was performed using Q5 for vector and insert amplification. The PCR conditions were: 0.02 U/μL polymerase, 0.5 μM each primer, 200 μM dNTPs, and 1 ng prelinearized template DNA. According to the following protocol: 98 °C (30 s) → [98 °C (10 s) → 60 °C (30 s) → 72 °C (2500 bp/min, 20 cycles for vector, 30 cycles for insert)] → 72 °C (2 min) → 10 °C (hold). After amplification, the PCR products were purified and recovered for further use. Additionally, 20 μL PCR was performed using Taq PCR Master Mix for each colony PCR following the manufacturer’s instructions. The corresponding PCR condition was: 95 °C (30 s) → [95 °C (15 s) → 55 °C (15 s) → 72 °C (1500 bp/min, 25 cycles)] → 72 °C (2 min) → 10 °C (hold). Agarose gel electrophoresis was used for identifying positive clones. Colonies that showed the same band as the insert were considered positive.

### Transformation evaluation

Unless otherwise stated, a 1 μL (4 μL for BL21(DE3)) mixture of linearized vector and insert was transformed into 25 μL (100 μL for BL21(DE3)) of competent cells, and then the mixture was first incubated for 25 min on ice, followed by heat shock at 42 °C for 60 s and transferred to ice for 2 min. After adding 1000 μL of LB medium, cells were subsequently incubated at 37 °C and shaking vigorously (220 rpm) for 45 min. After incubation, cells were centrifuged at 6000 rpm (~ 2500 g) for 1 min, pellets were resuspended in 75 μL of fresh LB medium then spread onto LB agar plates containing antibiotics and IPTG (only for pUC19-based plasmids that express fluorescent protein) for 16 h at 37 °C. Colonies were manually counted, and the positive ratio was assessed by colony PCR (with the “UMCS-Seq-F + UMCS-Seq-R” primer pair) or counting under a 485 nm light source through a 535 nm filter after an additional incubation at 4 °C for 48 h. Part of the positive clones were further confirmed by Sanger sequencing (Sangon Biotech).

### Data analyses

Data are expressed as the mean ± SD from four or more experiments. Statistical analyses were performed using one-way ANOVA with the comparison methods of the Tukey test (equal variances) or the Games-Howell test (unequal variances). Data sharing a letter in the same group were not considered to be significantly different at the 5% level.

## Supplementary Information

**Additional file 1 Table S1**. An overview of some existing methods developed for plasmid cloning.

**Additional file 2 Table S2**. A selection of UMCS based cloning for XL-10 Gold and JM109(DE3).

**Additional file 3 Table S3**. List of the primers and oligonucleotides used throughout this study.

**Additional file 4 Figure S1**. Effects of insert/vector ratio on assembly efficiency for vector linearized by *Eco*RV digestion.

**Additional file 5 Figure S2**. Assumed mechanism of bacterial in vivo assembly with nonhomologous regions.

**Additional file 6 Figure S3**. Efficiency evaluation of UMCS based subcloning for Top10 and BL21(DE3).

**Additional file 7.** Supplementary image file. Vector format of Figure S1, S2, S5.

**Additional file 8.** Supplementary image file. Uncropped original image of Fig. 6c.

**Additional file 9.** Supplementary image file. Uncropped original image of Fig. 6g.

**Additional file 10.** Supplementary data file. Raw data points as used for Figs. 4, 5, 6, 7, 8, S1, S3.

**Additional file 11.** Supplementary protocol description. Standard protocols for UMCS based cloning.

## Data Availability

The datasets used and analyzed in the current study are available from the corresponding author on reasonable request or as in supplementary files.

## Abbreviations

MCS: Multiple Cloning Site
PCR: Polymerase Chain Reaction
IVA: In Vivo Assembly
ELIC: Exonuclease and Ligation-Independent Cloning
LIC: Ligation-Independent Cloning
SLIC: Sequence and Ligation-Independent Cloning
HAC: Homologous Alignment Cloning
SLiCE: Seamless Ligation Cloning Extract
IPTG: Isopropyl β-D-1-thiogalactopyranoside
EGFP: Enhanced Green Fluorescent Protein
RXRα: Retinoid X Receptor Alpha
p85α: Phosphoinositide 3-kinase p85α
RLuc: Renilla Luciferase
LB medium: Luria-Bertani medium
