# Supplementary material for: Simplified plasmid cloning with a universal MCS design and bacterial in vivo assembly
Source: BMC Biotechnol. 2021 Mar 15;21:24. doi: 10.1186/s12896-021-00679-6 (PMC7962268; doi:10.1186/s12896-021-00679-6)
Supplement: Supplementary file 1 — Additional file 1 Table S1. An overview of some existing methods developed for plasmid cloning. [file 12896_2021_679_MOESM1_ESM.docx]

Table S1 An overview of some existing methods developed for plasmid cloning

| Method Name | Method Description | Advantage | Disadvantage | Reference |
| --- | --- | --- | --- | --- |
| IVA Cloning | Homologous recombination based, *recA*-independent *in vivo* assembly method for all cloning procedures (insertions, deletions, site-directed mutagenesis and subcloning) mediated by PCR. | 1. Eliminates the need for enzymatic assembly and reduces all molecular cloning procedures to a single-tube, single-step PCR.  2. The method is efficient, seamless and sequence-independent. | 1. *Dpn*I is needed for template digestion.  2. PCR amplified vectors might contain random mutation(s).  3. The molar ratio of insert/vector is not easy to control.  4. Primers for the amplification of insert will have to be redesigned and synthesized according to the restriction enzymes used for vectors not amenable to PCR amplification. | [6] |
| Fast Cloning | An efficient PCR-based cloning technique to insert any DNA fragment with ~16 bp overlapping regions into a plasmid vector or a gene (cDNA) in a vector at any desired position. | 1. Eliminates the need for PCR purification/gel purification kit and cloning kit.  2. It is a ligation-independent seamless cloning method. | 1. *Dpn*I is needed for template digestion.  2. PCR amplified vectors might contain random mutation(s).  3. Not suitable for PCR-resistant vectors. | [7] |
| ELIC | A homologous recombinatorial cloning method uses a mixture of purified linear dsDNA molecules with homologous ends for direct transformation. | 1. No incubation or enzyme treatments are needed for creating recombinants from linear fragments.  2. Vectors are linearized by restriction digestion without the possibilities of random mutation(s) introduced by PCR. | 1. Double digestion and subsequently gel-purification are required for vector linearization.  2. Primers for the amplification of insert will have to be redesigned and synthesized according to the restriction enzymes used for vector linearization. | [8] |

Table S1 An overview of some existing methods developed for plasmid cloning *(Continued)*

| Method Name | Method Description | Advantage | Disadvantage | Reference |
| --- | --- | --- | --- | --- |
| SLIC | Sequence and ligation–independent cloning, which mimics *in vivo* homologous recombination by relying on exonuclease-generated ssDNA overhangs in the insert and vector fragments, and the assembly of these fragments by recombination *in vitro*. | 1. Transformation efficiency is high enough to allow the assembly of up to 10 fragments simultaneously with a nearly 20% positive ratio.  2. Functions more efficiently at low DNA concentrations when combined with RecA to catalyze homologous recombination. | 1. T4 DNA polymerase treatment is necessary for the method, which causes additional time and reagents.  2. *Dpn*I is needed for template digestion.  3. Primers for the amplification of insert will have to be redesigned and synthesized according to the restriction enzymes used for vector linearization.  4. Overhangs with a length of >20 bp are required for effective transformation. | [9] |
| HAC | An optimized version of SLIC utilizing the 3′ to 5′ exonuclease activity of T4 DNA polymerase, creates overlapping homologous DNA on each molecule. Then the exonuclease activity of T4 polymerase is quenched by the addition of EDTA, and the following annealing step ensures high yield and high fidelity vector formation. | 1. Preparation of transformation can be done within 2 min, much faster than the original SLIC.  2. Overhang length of ~15 bp is enough for regular subcloning. | 1. T4 DNA polymerase treatment is necessary for the method, which causes additional steps and reagents.  2. Primers for the amplification of insert will have to be redesigned and synthesized according to the restriction enzymes used for vector linearization. | [10] |

Table S1 An overview of some existing methods developed for plasmid cloning *(Continued)*

| Method Name | Method Description | Advantage | Disadvantage | Reference |
| --- | --- | --- | --- | --- |
| One-step SLIC | A modified version of SLIC utilizes only T4 DNA polymerase for high efficiency and directional cloning. The whole process can be achieved by direct bacterial transformation 2.5 min after mixing any linearized vector, an insert(s) prepared by PCR, and T4 DNA polymerase in a tube at room temperature. | Preparation of transformation can be done within 12.5 min, faster than the original SLIC. | 1. T4 DNA polymerase treatment is necessary for the method, which causes additional steps and reagents.  2. *Dpn*I is needed for template digestion when vector is linearized by PCR.  3. Primers for the amplification of insert will have to be redesigned and synthesized according to the restriction enzymes used for vector linearization. | [11] |
| In-Fusion Cloning | A single-reaction method designed for fast, directional assembling of one or more DNA molecules with ~15 bp overlaps by the action of a 3′ exonuclease. | 1. Efficiently and seamlessly clone a broad range of fragment sizes.  2. Multiple DNA fragments can be assembled simultaneously into any vector in a single reaction. | 1. The cost of this method is relatively high compared to conventional methods.  2. Competent cell strains such as Top10, DH10B, and MC1061 are not recommended because the In-Fusion products might not be stable within these cells. | [12] |
| Gateway Cloning | Gateway is based on the recombination reactions that mediate the integration and excision of phage λ into and from the *E. coli* genome, respectively. The technology contains two different reactions named “BP” and “LR” that were used to generate “entry clone” and “expression clone”, respectively. | 1. The DNA fragment within entry clone can be moved across any expression system in just one recombination step without further sequencing.  2. The positive (antibiotic) and negative (CcdB) selection markers used in Gateway Cloning significantly improved cloning accuracy. | 1. As a commercial cloning kit, this method’s cost is relatively high compared to conventional methods such as restriction-ligation cloning.  2. Dedicated vectors with the specific sequence for recombination is necessary for the reactions, which eventually left “scars” in final products. | [13] |

Table S1 An overview of some existing methods developed for plasmid cloning *(Continued)*

| Method Name | Method Description | Advantage | Disadvantage | Reference |
| --- | --- | --- | --- | --- |
| SLiCE | Seamless ligation cloning extract (SLiCE) cloning uses the endogenous recombination activity of *E. coli* cellular extracts *in vitro* to ligate insert and vector DNA fragments. Then the ligated DNA can be transformed at high efficiency. | 1. Transformation efficiency and fidelity are both satisfied with this method.  2. Insert fragments can be assembled into restriction enzyme-digested vectors with flanking heterologous sequences. | 1. The preparation of SLiCE could be time-consuming.  2. The SLiCE reaction takes additional time during transformation preparation. | [14,15] |
| Gibson Assembly | An isothermal, single-reaction method for assembling multiple overlapping DNA molecules by the concerted action of a 5′ exonuclease, a DNA polymerase and a DNA ligase. The method can be used to seamlessly construct synthetic and natural genes, genetic pathways and entire genomes. | 1. An ideal seamless method for complex assembly.  2. Highly effective for regular subcloning or multi-fragment assembly. | 1. The cost of this method is relatively high compared to conventional methods.  2. Not suitable for the assembly of short fragments (e.g., <100 bp). | [16] |
| Golden Gate Assembly | An assembly protocol based on the use of type IIs restriction enzymes, which allows the conversion of more than half of all input plasmids into the desired recombinant product in a short time (usually less than 30 min). | 1. Ideal for simultaneous and directional assembly of multiple fragments in one seamless reaction.  2. Both digestion and ligation steps are carried out simultaneously in a single-tube with high cloning efficiency. | 1. The cost of this method is relatively high compared to conventional methods.  2. The Type IIs site used in the reaction must not be present within the fragments for assembling. | [17] |
