# Supplementary material for: Simplified plasmid cloning with a universal MCS design and bacterial in vivo assembly
Source: BMC Biotechnol. 2021 Mar 15;21:24. doi: 10.1186/s12896-021-00679-6 (PMC7962268; doi:10.1186/s12896-021-00679-6)
Supplement: Supplementary file 2 — Additional file 2 Table S2. A selection of UMCS based cloning for XL-10 Gold and JM109(DE3). [file 12896_2021_679_MOESM2_ESM.docx]

Table S2 A selection of UMCS based cloning for XL-10 Gold and JM109(DE3)

| Vector | Linearize  Method | Insert | *E. coli* Strain | Colony Count (estimated) | Positive Ratio^a^ |
| --- | --- | --- | --- | --- | --- |
| pCDNA3.1(+)-UMCS (5390 bp, 17 ng) | *Eco*RV Digestion | Nurr77 CDS (1797 bp, 28 ng) | XL-10 Gold | >1000 | 3/6 |
| pCDNA3.1(+)-UMCS (5390 bp, 17 ng) | *Eco*RV Digestion | RARα CDS (1389 bp, 22 ng) | XL-10 Gold | >1000 | 2/6 |
| pBind-UMCS  (6372 bp, 20 ng) | *Sal*I Digestion | p85α (2175 bp, 41 ng) | XL-10 Gold | 500~1000 | 3/12 |
| pET24a(+)-UMCS (5312 bp, 17 ng) | PCR | Nurr77 CDS (1797 bp, 28 ng) | XL-10 Gold | 500~1000 | 3/3 |
| pET24a(+)-UMCS (5312 bp, 17 ng) | PCR | Nurr77 CDS (1797 bp, 28 ng) | JM109(DE3) | 100~200 | 3/3 |
| pET24a(+)-UMCS (5312 bp, 17 ng) | *Sal*I Digestion | RARα CDS (1389 bp, 22 ng) | XL-10 Gold | >1000 | 5/12 |
| pET24a(+)-UMCS (5312 bp, 17 ng) | *Sal*I Digestion | RARα CDS (1389 bp, 22 ng) | JM109(DE3) | 100~200 | 2/12 |
| pET15b-UMCS (5739 bp, 20 ng) | *Sal*I Digestion | RXRα-LBD CDS (720 bp, 13 ng) | XL-10 Gold | >1000 | 4/12 |
| pET15b-UMCS (5739 bp, 20 ng) | *Sal*I Digestion | RXRα-LBD CDS (720 bp, 13 ng) | JM109(DE3) | 200~500 | 4/12 |

a: Positive ratio was calculated by positives colonies/total colonies verified.
