# Supplementary material for: Simplified plasmid cloning with a universal MCS design and bacterial in vivo assembly
Source: BMC Biotechnol. 2021 Mar 15;21:24. doi: 10.1186/s12896-021-00679-6 (PMC7962268; doi:10.1186/s12896-021-00679-6)
Supplement: Supplementary file 3 — Additional file 3 Table S3. List of the primers and oligonucleotides used throughout this study. [file 12896_2021_679_MOESM3_ESM.docx]

Table S3 List of the primers and oligonucleotides used throughout this study

| Primer Name | Primer Description | Primer Sequence (5′→3′) |
| --- | --- | --- |
| UMCS-PCR-F | Forward primer with 15-bp linker for UMCS vector amplification | ACAAGCGACGTCACC |
| UMCS-PCR-R | Reverse primer with 15-bp linker for UMCS vector amplification | AGTGCTGCCTTCGTC |
| UMCS-PCR-S18-F | Forward primer with 18-bp linker for UMCS vector amplification | *GAC*ACAAGCGACGTCACC |
| UMCS-PCR-S18-R | Reverse primer with 18-bp linker for UMCS vector amplification | *GAC*AGTGCTGCCTTCGTC |
| UMCS-Seq-F | Forward primer for UMCS colony PCR | GACGAAGGCAGCACT |
| UMCS-Seq-R | Reverse primer for UMCS colony PCR | GGTGACGTCGCTTGT |
| pUC-UMCS-S-F | Forward primer for UMCS with *Sal*I recognition site cloning into pUC19 | CACT*GTCGAC*ACAAGCGACGTCACC*CTCGAG*TTAAGCCAGCCCCGAC |
| pUC-UMCS-S-R | Reverse primer for UMCS with *Sal*I recognition site cloning into pUC19 | GT*GTCGAC*AGTGCTGCCTTCGTCCATAGCTGTTTCCTGTGTGAAATTG |
| pUC-UMCS-E-F | Forward primer for UMCS with *Eco*RV recognition site cloning into pUC19 | CACT*GATATC*ACAAGCGACGTCACC*CTCGAG*TTAAGCCAGCCCCGAC |
| pUC-UMCS-E-R | Reverse primer for UMCS with *Eco*RV recognition site cloning into pUC19 | GT*GATATC*AGTGCTGCCTTCGTCCATAGCTGTTTCCTGTGTGAAATTG |
| pET24-UMCS-BXh-F | Forward oligo for UMCS cloning into pET24a(+) | *GATCC*GACGAAGGCAGCACT*GTCGAC*ACAAGCGACGTCACC*C* |
| pET24-UMCS-BXh-R | Reverse oligo for UMCS cloning into pET24a(+) | *TCGAG*GGTGACGTCGCTTGT*GTCGAC*AGTGCTGCCTTCGTC*G* |
| pAB-UMCS-BXb-F | Forward oligo for UMCS cloning into pACT and pBind | *GATC*GACGAAGGCAGCACT*GTCGAC*ACAAGCGACGTCACC*T* |
| pAB-UMCS-BXb-R | Reverse oligo for UMCS cloning into pACT and pBind | *CTAGA*GGTGACGTCGCTTGT*GTCGAC*AGTGCTGCCTTCGTC |
| pCold-UMCS-NXb-F | Forward oligo for UMCS cloning into pCold TF DNA | *TATG*GACGAAGGCAGCACT*GTCGAC*ACAAGCGACGTCACC*T* |
| pCold-UMCS-NXb-R | Reverse oligo for UMCS cloning into pCold TF DNA | *CTAGA*GGTGACGTCGCTTGT*GTCGAC*AGTGCTGCCTTCGTC*CA* |
| EGFP-Xh-F | Forward primer for EGFP cloning into pUC19-UMCS | GAA*CTCGAG*ATGGTGAGCAAGGGCGAG |

Table S3 List of the primers and oligos used throughout this study *(Continued)*

| Primer Name | Primer Description | Primer Sequence (5′→3′) |
| --- | --- | --- |
| EGFP-Xh-R | Reverse primer for EGFP cloning into pUC19-UMCS | GAA*CTCGAG*TTACTTGTACAGCTCGTCC |
| mCY-UMCS-S6-F | Forward primer with 6-, 9-, 12-, 15-, 18-bp linker (with 3 bp of *Sal*I recognition site) for mCherry cloning into pUC19-UMCS-S-EGFP | ACT*GTC*ATGGTGAGCAAGGGCGAG |
| mCY-UMCS-S9-F |  | AGCACT*GTC*ATGGTGAGCAAGGGCGAG |
| mCY-UMCS-S12-F |  | GGCAGCACT*GTC*ATGGTGAGCAAGGGCGAG |
| mCY-UMCS-S15-F |  | GAAGGCAGCACT*GTC*ATGGTGAGCAAGGGCGAG |
| mCY-UMCS-S18-F |  | GACGAAGGCAGCACT*GTC*ATGGTGAGCAAGGGCGAG |
| mCY-UMCS-S6-R | Reverse primer with 6-, 9-, 12-, 15-, 18-bp linker (with 3 bp of *Sal*I recognition site) for mCherry cloning into pUC19-UMCS-S-EGFP | TGT*GTC*TTACTTGTACAGCTCGTCC |
| mCY-UMCS-S9-R |  | GCTTGT*GTC*TTACTTGTACAGCTCGTCC |
| mCY-UMCS-S12-R |  | GTCGCTTGT*GTC*TTACTTGTACAGCTCGTCC |
| mCY-UMCS-S15-R |  | GACGTCGCTTGT*GTC*TTACTTGTACAGCTCGTCC |
| mCY-UMCS-S18-R |  | GGTGACGTCGCTTGT*GTC*TTACTTGTACAGCTCGTCC |
| mCY-UMCS-E9-F | Forward primer with 9-, 12-, 15-, 18-bp linker (with 3 bp of *Eco*RV recognition site) for mCherry cloning into pUC19-UMCS-E-EGFP | AGCACT*GAT*ATGGTGAGCAAGGGCGAG |
| mCY-UMCS-E12-F |  | GGCAGCACT*GAT*ATGGTGAGCAAGGGCGAG |
| mCY-UMCS-E15-F |  | GAAGGCAGCACT*GAT*ATGGTGAGCAAGGGCGAG |
| mCY-UMCS-E18-F |  | GACGAAGGCAGCACT*GAT*ATGGTGAGCAAGGGCGAG |
| mCY-UMCS-E9-R | Reverse primer with 9-, 12-, 15-, 18-bp linker (with 3 bp of *Eco*RV recognition site) for mCherry cloning into pUC19-UMCS-E-EGFP | GCTTGT*GAT*TTACTTGTACAGCTCGTCC |
| mCY-UMCS-E12-R |  | GTCGCTTGT*GAT*TTACTTGTACAGCTCGTCC |
| mCY-UMCS-E15-R |  | GACGTCGCTTGT*GAT*TTACTTGTACAGCTCGTCC |
| mCY-UMCS-E18-R |  | GGTGACGTCGCTTGT*GAT*TTACTTGTACAGCTCGTCC |
| mCY-UMCS-9-F | Forward primer with 9-, 12-bp linker for mCherry cloning into pUC19-UMCS-S-EGFP and pUC19-UMCS-E-EGFP | GGCAGCACTTGGTGAGCAAGGGCGAG |
| mCY-UMCS-12-F |  | GAAGGCAGCACTTGGTGAGCAAGGGCGAG |

Table S3 List of the primers and oligos used throughout this study *(Continued)*

| Primer Name | Primer Description | Primer Sequence^a^ (5′→3′) |
| --- | --- | --- |
| mCY-UMCS-15-F | Forward primer with 15-bp linker for mCherry cloning into pUC19-UMCS-S-EGFP and pUC19-UMCS-E-EGFP | GACGAAGGCAGCACTTGGTGAGCAAGGGCGAG |
| mCY-UMCS-9-R | Reverse primer with 9-, 12-, 15-bp linker for mCherry cloning into pUC19-UMCS-S-EGFP and pUC19-UMCS-E-EGFP | GTCGCTTGTTTACTTGTACAGCTCGTCC |
| mCY-UMCS-12-R |  | GACGTCGCTTGTTTACTTGTACAGCTCGTCC |
| mCY-UMCS-15-R |  | GGTGACGTCGCTTGTTTACTTGTACAGCTCGTCC |
| RXR-UMCS-15-F | Forward primer with 15-bp linker for RXRα cloning into vectors with UMCS | GACGAAGGCAGCACTATGGACACCAAACATTTCCTG |
| RXR-UMCS-15-R | Reverse primer with 15-bp linker for RXRα cloning into vectors with UMCS | GGTGACGTCGCTTGTCTAAGTCATTTGGTGCGGC |
| p85-UMCS-15-F | Forward primer with 15-bp linker for p85α cloning into vectors with UMCS | GACGAAGGCAGCACTATGAGTGCTGAGGGGTAC |
| p85-UMCS-15-R | Reverse primer with 15-bp linker for p85α cloning into vectors with UMCS | GGTGACGTCGCTTGTTCATCGCCTCTGCTGTG |
| mCY-RXR-18-R | Reverse primer with 18-bp linker for *in vivo* assembly of mCherry and RXRα | GAAATGTTTGGTGTCCATTTACTTGTACAGCTCGTCC |
| RXR-F | Forward primer for RXRα amplification | ATGGACACCAAACATTTCCTG |
| RXR-R | Reverse primer for RXRα amplification | CTAAGTCATTTGGTGCGGC |
| RLuc-RXR-18-F | Forward primer with 18-bp linker for *in vivo* assembly of RXRα and RLuc | CCGCACCAAATGACTTAGATGACTTCGAAAGTTTATG |
| RLuc-UMCS-15-R | Reverse primer with 15-bp linker for *in vivo* assembly of RLuc | GGTGACGTCGCTTGTTTATTGTTCATTTTTGAG |

a: Restriction endonuclease recognition sequences (or part of them) within primers are marked as italic and red, while the homologous linker regions are represented by underlined．
