# Supplementary material for: Simplified plasmid cloning with a universal MCS design and bacterial in vivo assembly
Source: BMC Biotechnol. 2021 Mar 15;21:24. doi: 10.1186/s12896-021-00679-6 (PMC7962268; doi:10.1186/s12896-021-00679-6)
Supplement: Supplementary file 4 — Additional file 4 Figure S1. Effects of insert/vector ratio on assembly efficiency for vector linearized by EcoRV digestion. [file 12896_2021_679_MOESM4_ESM.docx]

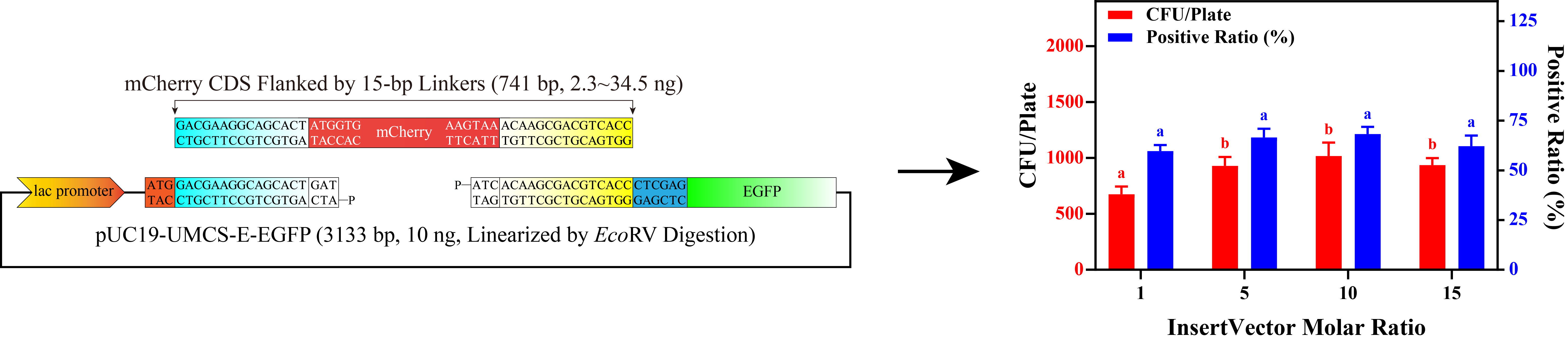


**Fig. S1** Effects of insert/vector ratio on assembly efficiency for vector linearized by *Eco*RV digestion. Effects of insert/vector molar ratio on the recombination of pUC19-UMCS-E-EGFP linearized by *Eco*RV digestion and the mCherry sequence flanked by 15-bp homologous linkers (n=4).
