# Supplementary material for: Simplified plasmid cloning with a universal MCS design and bacterial in vivo assembly
Source: BMC Biotechnol. 2021 Mar 15;21:24. doi: 10.1186/s12896-021-00679-6 (PMC7962268; doi:10.1186/s12896-021-00679-6)
Supplement: Supplementary file 5 — Additional file 5 Figure S2. Assumed mechanism of bacterial in vivo assembly with nonhomologous regions. [file 12896_2021_679_MOESM5_ESM.docx]

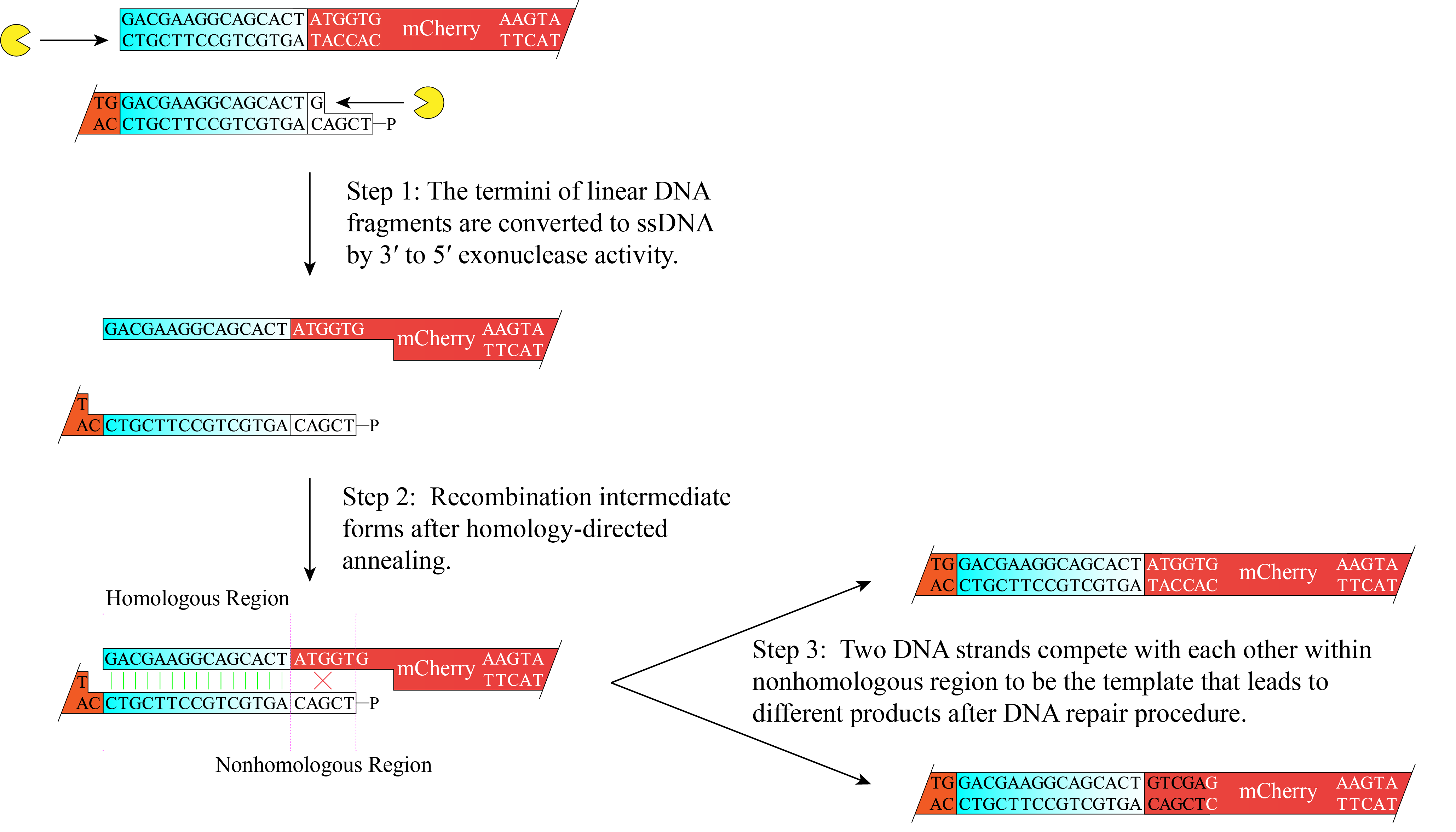


**Fig. S2** Assumed mechanism of bacterial *in vivo* assembly with nonhomologous regions. Dedicated vector was first digested by *Sal*I (as an example), then co-transformed into the bacterial cell with mCherry CDS (as an example) including Linker-F sequence at its 5' end. Through step 1~3, two possible plasmid products can be identified at different frequencies.
