# Supplementary material for: Simplified plasmid cloning with a universal MCS design and bacterial in vivo assembly
Source: BMC Biotechnol. 2021 Mar 15;21:24. doi: 10.1186/s12896-021-00679-6 (PMC7962268; doi:10.1186/s12896-021-00679-6)
Supplement: Supplementary file 6 — Additional file 6 Figure S3. Efficiency evaluation of UMCS based subcloning for Top10 and BL21(DE3). [file 12896_2021_679_MOESM6_ESM.docx]

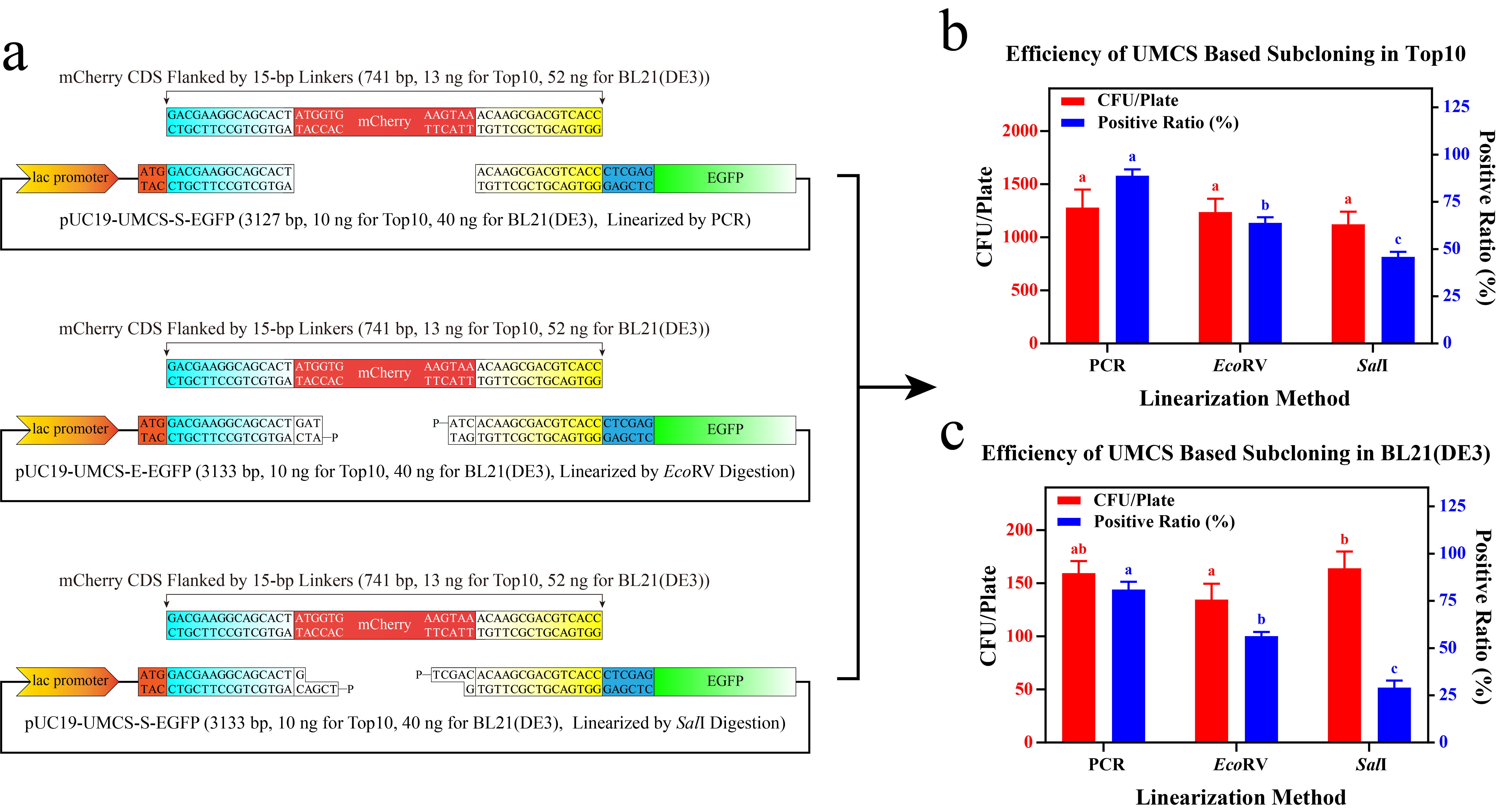


**Fig. S3** Efficiency evaluation of UMCS based subcloning for Top10 and BL21(DE3). Dedicated vectors were first linearized (**a**) by PCR, *Eco*RV digestion, or *Sal*I digestion, then co-transformed into Top10 (**b**) and BL21(DE3) (**c**) chemically competent cells with the mCherry sequence flanked by 15-bp homologous linkers (n=4).
