# Supplementary figures and images for: Simplified plasmid cloning with a universal MCS design and bacterial in vivo assembly
Source: BMC Biotechnol. 2021 Mar 15;21:24. doi: 10.1186/s12896-021-00679-6 (PMC7962268; doi:10.1186/s12896-021-00679-6)

Figure S1

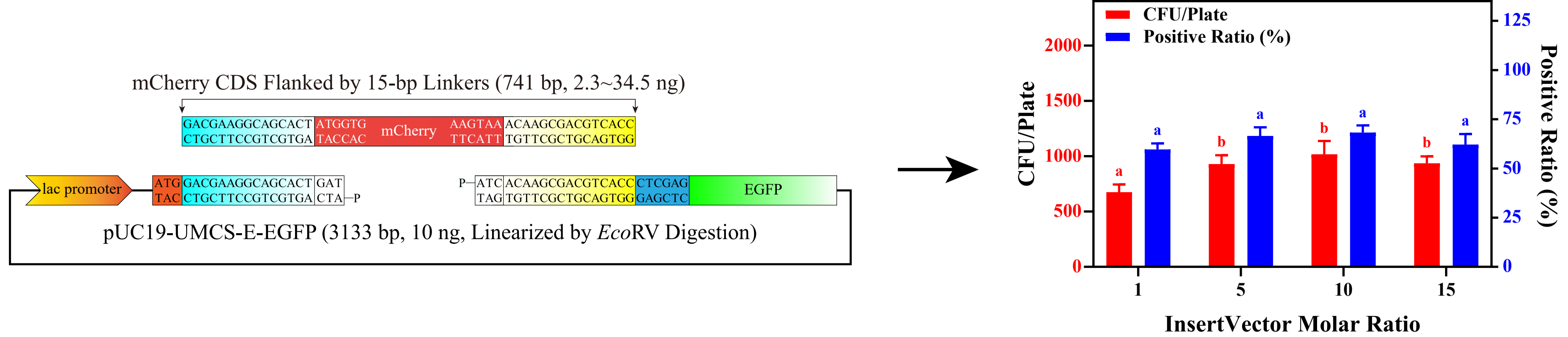

Figure S2

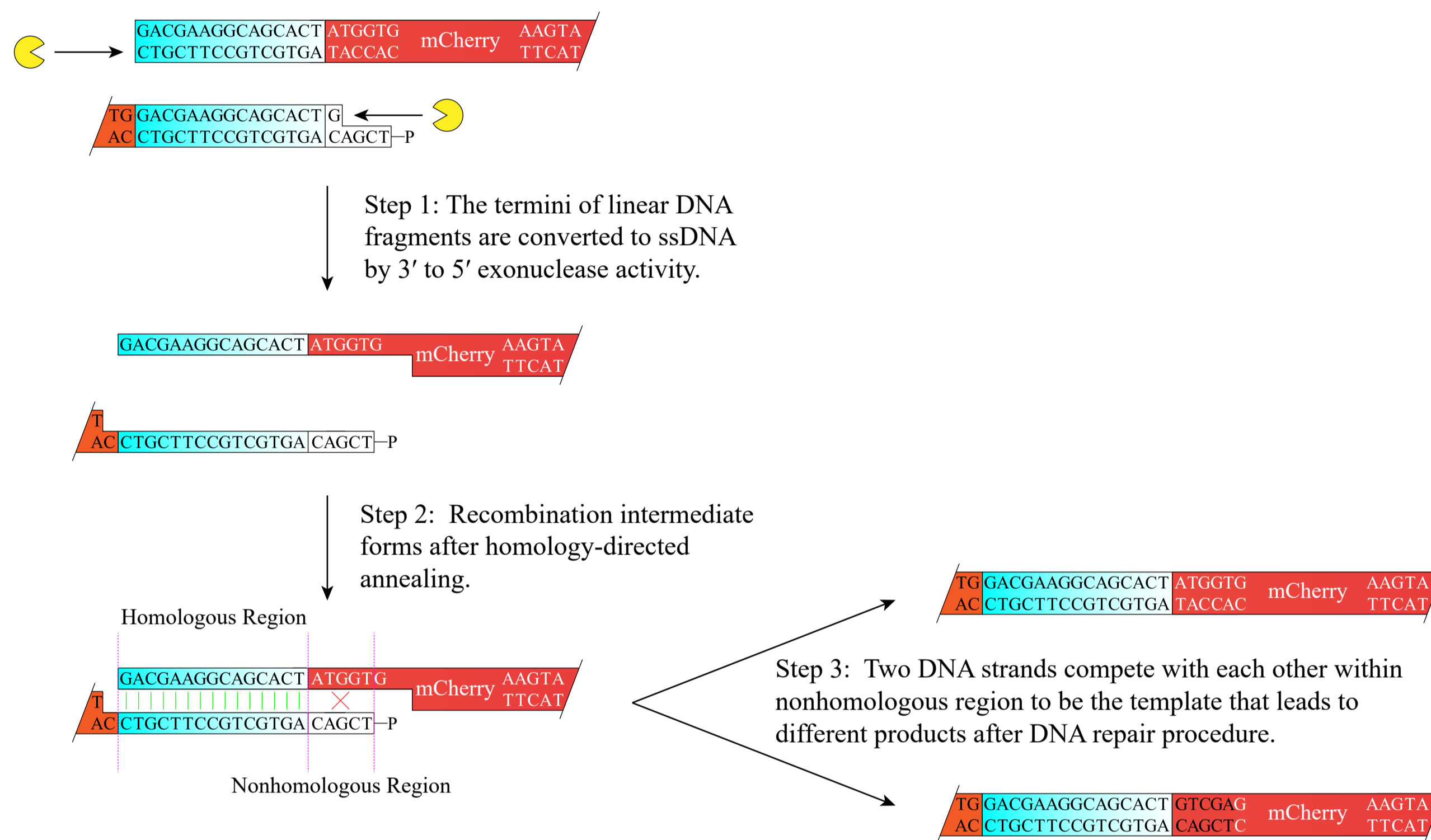

Figure S3

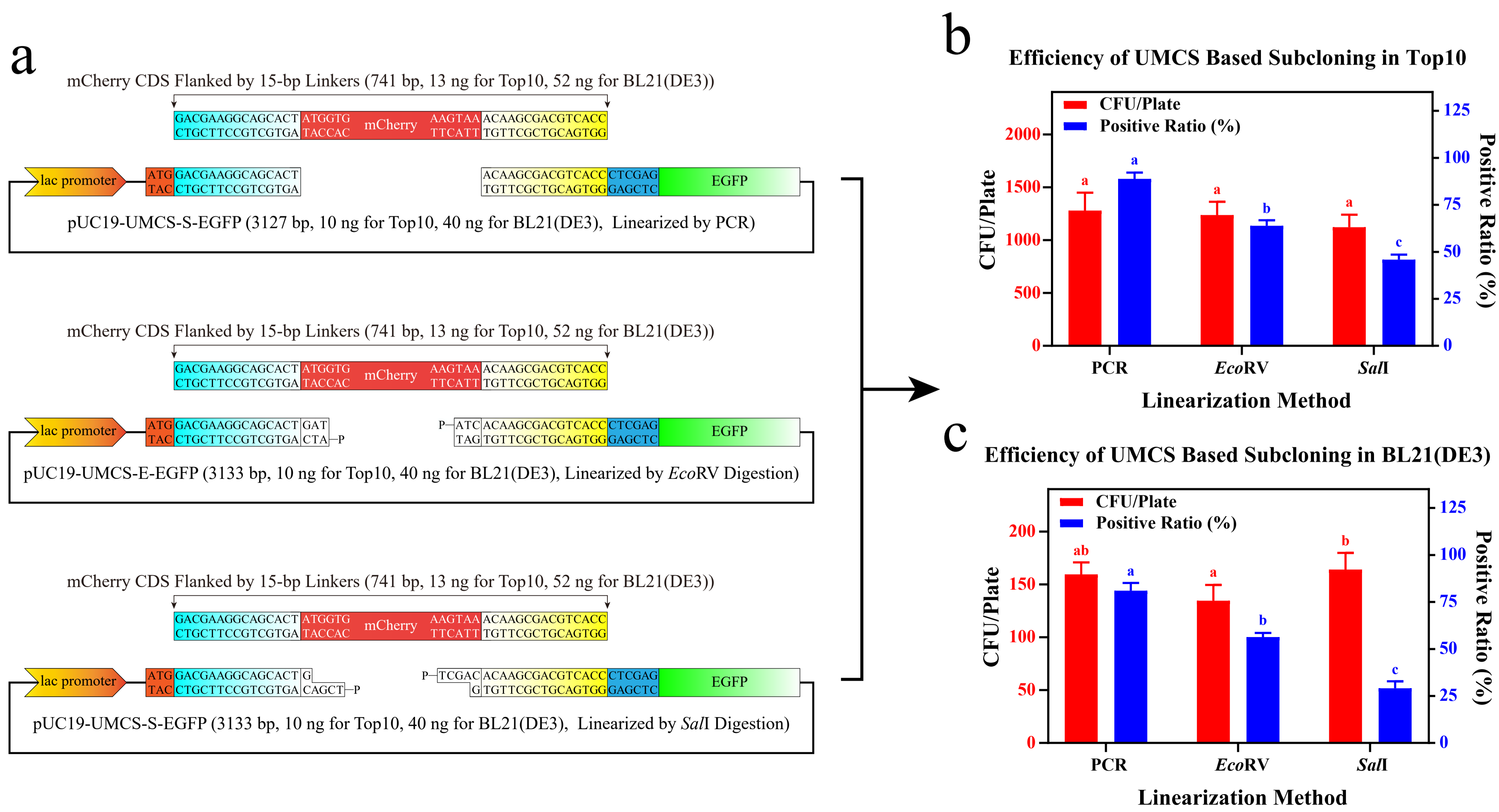

Supplement: Supplementary file 7 — Additional file 7. Supplementary image file. Vector format of Figure S1, S2, S5. [file 12896_2021_679_MOESM7_ESM.pdf]

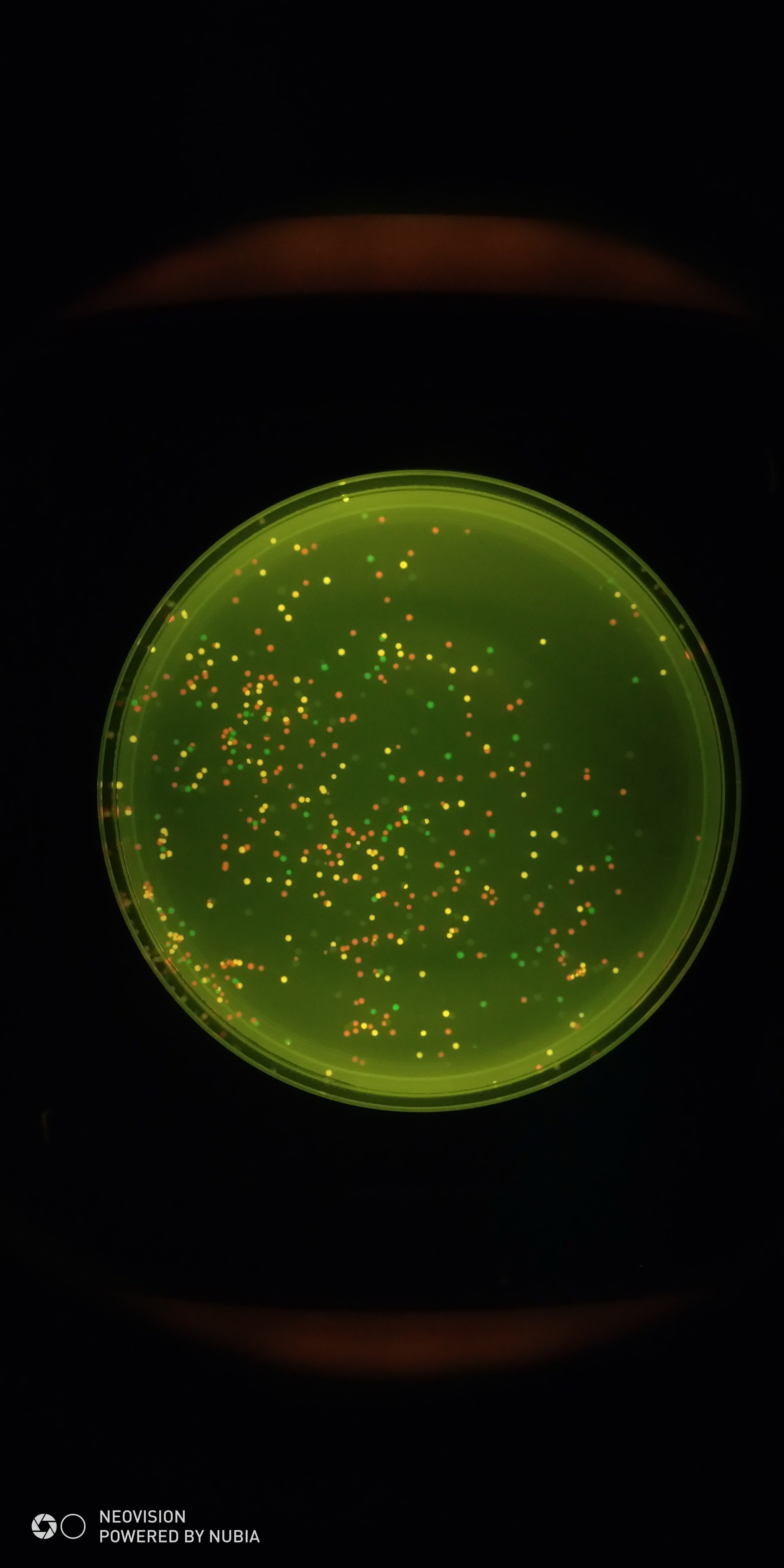

Supplement: Supplementary file 8 — Additional file 8. Supplementary image file. Uncropped original image of Fig. 6c. [file 12896_2021_679_MOESM8_ESM.jpg]

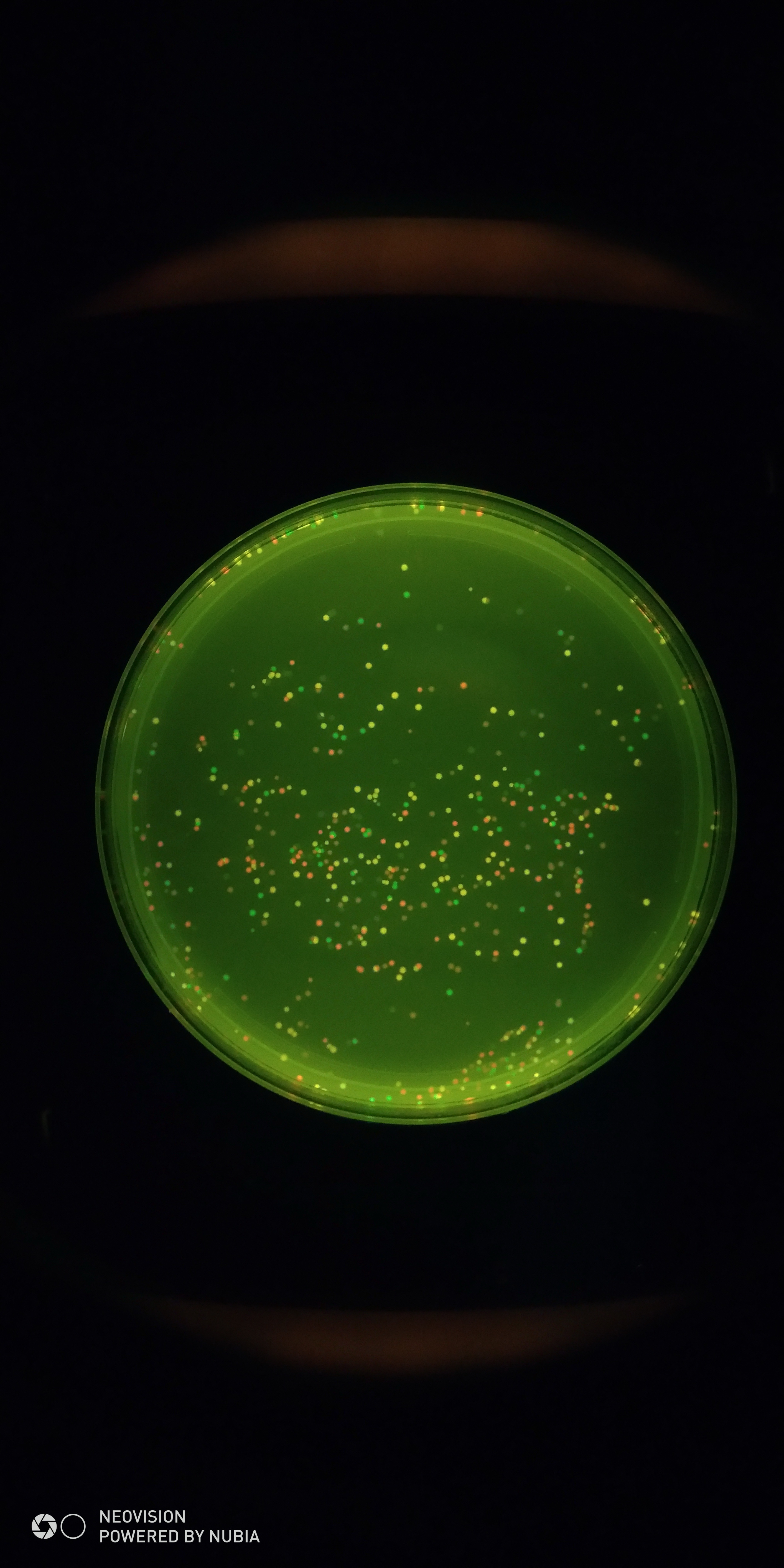

Supplement: Supplementary file 9 — Additional file 9. Supplementary image file. Uncropped original image of Fig. 6g. [file 12896_2021_679_MOESM9_ESM.jpg]
