# Supplementary material for: Simplified plasmid cloning with a universal MCS design and bacterial in vivo assembly
Source: BMC Biotechnol. 2021 Mar 15;21:24. doi: 10.1186/s12896-021-00679-6 (PMC7962268; doi:10.1186/s12896-021-00679-6)
Supplement: Supplementary file 11 — Additional file 11. Supplementary protocol description. Standard protocols for UMCS based cloning. [file 12896_2021_679_MOESM11_ESM.docx]

**Standard protocols for UMCS based cloning**

Part I: Vector Linearization:

1. By Restriction Digestion^a^:
2. Combine the following reaction components to the final volume of 20 μL at room temperature in the order indicated:

Water (variable amount) + Reaction Buffer (2 μL) + Vector DNA (up to 1 μg) + Enzyme (1 μL)

1. Mix gently and spin down.
2. Incubate at 37°C for 15 min.
3. Recover the digested vector by PCR Product Purification Kit directly or by DNA Gel Extraction Kit after agarose gel electrophoresis^b^.
4. By PCR^c^:
5. Combine the following reaction components to the final volume of 50 μL on ice in the order indicated:

Water (variable amount) + Reaction Buffer (10 μL) + 10 mM dNTPs (1 μL) +10 µM Forward Primer (2.5 μL) + 10 µM Reverse Primer (2.5 μL) + Template DNA (0.1~0.5 ng/1000 bp of template length)^d^ + DNA Polymerase (0.5 μL).

1. Gently mix the reaction then transfer tubes to a thermocycler and begin thermocycling with the following PCR conditions: 98°C (30 s) →[98°C (10 s) →60°C (30 s) →72°C (2500 bp/min, 20 cycles)]→72°C (2 min) →10°C (hold).
2. After amplification, the PCR products were purified and recovered by PCR Product Purification Kit for further use.

a: Protocol for FastDigest *Sal*I is listed here as an example. Please refer to the manufacturer’s instructions for detailed experiment conditions.

b: Purification by agarose gel electrophoresis will further reduce transformation background, but it is not a necessary step.

c: Protocol for NEB Q5 was listed here as an example. Please refer to the manufacturer’s instructions for detailed experiment conditions.

d: Digested vector DNA from Protocol 1 is recommended as the template DNA for background reduction.

Part II: Insert Amplification^a^:

1. Combine the following reaction components to the final volume of 50 μL on ice in the order indicated:

Water (variable amount) + Reaction Buffer (10 μL) + 10 mM dNTPs (1 μL) +10 µM Forward Primer (2.5 μL) + 10 µM Reverse Primer (2.5 μL) + Template DNA (0.1~0.5 ng/1000 bp of template length)^b^ + DNA Polymerase (0.5 μL).

1. Gently mix the reaction then transfer tubes to a thermocycler and begin thermocycling with the following PCR conditions: 98°C (30 s) →[98°C (10 s) →60°C (30 s) →72°C (2500 bp/min, 30 cycles)]→72°C (2 min) →10°C (hold).
2. After amplification, the PCR products were purified and recovered by PCR Product Purification Kit for further use.

a: Protocol for NEB Q5 is listed here as an example. Please refer to the manufacturer’s instructions for detailed experiment conditions.

b: Linearized template is recommended for background reduction.

Part III: Colony PCR^a^:

1. Pick one colony with a 10 μL pipet tip, then resuspend the cells in 10 μL LB medium containing specified antibiotic by pipetting.
2. Combine the following reaction components to the final volume of 20 μL at room temperature in the order indicated:

Water (variable amount) + 10 µM Forward Primer (1 μL) + 10 µM Reverse Primer (1 μL) + Resuspended Cells (1 μL) + 2×Taq Master Mix (10 μL).

1. Gently mix the reaction then transfer tubes to a thermocycler and begin thermocycling with the following PCR conditions: 95°C (30 s) →[95°C (15 s) →55°C (15 s) →72°C (1500 bp/min, 25 cycles)]→72°C (2 min) →10°C (hold).
2. After amplification, agarose gel electrophoresis was used for identifying positive clones. Colonies that showed the same band as the insert were considered positive.

a: Protocol for 2×Taq PCR Master Mix is listed here as an example. Please refer to the manufacturer’s instructions for detailed experiment conditions.

Part IV: Transformation^a^:

1. Combine the linearized vector (final concentration: 1~5 ng/μL/1000 bp of vector length) and insert (5× molar concentration of vector) to the final volume of 2 μL at room temperature.
2. Transfer competent *E. coli* cells from a -80^o^C freezer to ice and wait 5 min to thaw the frozen cells.
3. Gently mix cells by tapping and aliquot 25 µL of cells for each transformation into a 1.5 mL centrifuge tube.
4. Add 1 μL of DNA solution from step 1 to one tube of aliquoted cells, then gently tap the tube to mix.
5. Incubated cells for 25 min on ice.
6. Heat-shock cells for 60 seconds in a 42°C water bath.
7. Place the cells on ice for 2 min.
8. Add 1 mL of LB medium, then incubated the cells at 37°C and shaking vigorously (220 rpm) for 45 min.
9. After incubation, cells were centrifuged at 6000 rpm (~2500 g) for 1 min. Pellet was resuspended in 75 μL of fresh LB medium then spread onto LB agar plate containing specified antibiotic.
10. Incubate the plate overnight at 37°C.

a: Protocol for heat shock transformation of DH5α is listed here as an example. Please refer to the manufacturer’s instructions for detailed experiment conditions.
